# Adverse drug reaction monitoring: support for pharmacovigilance at a tertiary care hospital in Northern Brazil

**DOI:** 10.1186/2050-6511-14-5

**Published:** 2013-01-08

**Authors:** Márcia Germana Alves de Araújo Lobo, Sandra Maria Botelho Pinheiro, José Gerley Díaz Castro, Valéria Gomes Momenté, Maria-Cristina S Pranchevicius

**Affiliations:** 1 Hospital Geral de Palmas, Av NS1, s/n Conj. 02 - Lote 01, Palmas, 77.054-970, Brasil; 2 Curso de Medicina, Universidade Federal do Tocantins, Av. NS 15 s/n (109 Norte), Palmas, 77001-090, Brasil; 3 Curso de Enfermagem, Universidade Federal do Tocantins, Av. NS 15 s/n (109 Norte), Palmas, 77001-090, Brasil; 4 Curso de Engenharia de Alimentos, Universidade Federal do Tocantins, Av. NS 15 s/n (109 Norte), Palmas, 77001-090, Brasil

## Abstract

**Background:**

Adverse drug reactions (ADRs) are recognised as a common cause of hospital admissions, and they constitute a significant economic burden for hospitals. Hospital-based ADR monitoring and reporting programmes aim to identify and quantify the risks associated with the use of drugs provided in a hospital setting. This information may be useful for identifying and minimising preventable ADRs and may enhance the ability of prescribers to manage ADRs more effectively. The main objectives of this study were to evaluate ADRs that occurred during inpatient stays at the Hospital Geral de Palmas (HGP) in Tocantins, Brazil, and to facilitate the development of a pharmacovigilance service.

**Methods:**

A prospective study was conducted at HGP over a period of 8 months, from January 2009 to August 2009. This observational, cross-sectional, descriptive study was based on an analysis of medical records. Several parameters were utilised in the data evaluation, including patient demographics, drug and reaction characteristics, and reaction outcomes. The reaction severity and predisposing factors were also assessed.

**Results:**

The overall incidence of ADRs in the patient population was 3.1%, and gender was not found to be a risk factor. The highest ADR rate (75.8%) was found in the adult age group 15 to 50 years, and the lowest ADR rate was found in children aged 3 to 13 years (7.4%). Because of the high frequency of ADRs in orthopaedic (25%), general medicine (22%), and oncology (16%) patients, improved control of the drugs used in these specialties is required. Additionally, the nurse team (52.7%) registered the most ADRs in medical records, most likely due to the job responsibilities of nurses. As expected, the most noticeable ADRs occurred in skin tissues, with such ADRs are more obvious to medical staff, with rashes being the most common reactions. Metamizole, tramadol, and vancomycin were responsible for 21, 11.6, and 8.4% of ADRs, respectively. The majority of ADRs had moderate severity (58.9%), thus requiring intervention. Type A reactions were the most common (82.1%). At least one predisposing factor was present in 79.9% of the reports examined, and the most common predisposing factor was polypharmacy.

**Conclusions:**

The results obtained will contribute to the development of strategies for the pharmacovigilance service at HGP and other hospitals throughout the country, which will improve the quality of ADR reporting and ensure safer drug use in Brazilian hospitals.

## Background

An adverse drug reaction (ADR) is defined by the World Health Organization (WHO) as any noxious, unintended, or undesired effect of a drug that occurs at doses used in humans for prophylaxis, diagnosis, or therapy [1]. ADRs are a major cause of morbidity and place a substantial burden on limited healthcare resources [2]. Multiple factors influence ADR susceptibility, including multiple drug therapy, disease severity, age, and the type and number of drugs prescribed [3-8]. Several studies have shown that the proportion of patients admitted with ADRs ranges from approximately 2.0 to 21.4%, whereas between 1.7 and 25.1% of inpatients are reported to have developed an ADR during their hospital stay [9-12]. There are marked differences in disease prevalence, access to medicines, drug use patterns, and drug management systems between developed and developing countries, and such differences impact the frequency and nature of ADRs [13].

Reports of ADRs have become an important component of monitoring and evaluation activities performed in hospitals [14]. This information may be useful for identifying and minimising preventable ADRs while generally enhancing the ability of prescribers to manage ADRs more effectively [15,16].

Few reports are available regarding the incidence of ADRs in Brazilian hospitals. The Hospital Geral de Palmas (HGP) is a highly complex, 220-bed, tertiary care reference centre and teaching hospital located in the city of Palmas in Northern Brazil. An ADR reporting program has existed in HGP since July 2001 and is coordinated by the Hospital’s Department of Pharmacy Practice. The ADR reporting unit of HGP is one of the reference centres of the National Pharmacovigilance Program.

The present study was undertaken to (1) determine the frequency of ADRs that occur in hospitalised patients and to classify the reactions according to the demographics of the affected patients and the preventability of the ADRs; (2) describe the types of drugs involved; (3) report the most common clinical manifestations associated with these ADRs and their severity; and (4) assess the predictive factors of ADRs.

## Methods

### Study design

A prospective study was conducted over a period of 8 months from January 2009 to August 2009 at HGP in Tocantins, Brazil. HGP is a 220-bed tertiary care hospital utilised by the entire state and its surrounding areas. The hospital’s specialties are general medicine and surgery. The study was observational, non-interventional, and based on the ADRs reported by multiple departments of HGP; the reports were coordinated by clinical pharmacists. Male and female inpatients, except those in the Intensive Care Unit and Emergency Room, were included in the study. HGP participates in standard pharmacovigilance and employs a system of spontaneous reporting, which was the form of reporting used in this study.

### Functioning of the ADR reporting system at HGP

There was no organised pharmacovigilance program at the hospital prior to the study. Clinical meetings with allied hospital healthcare professionals raised awareness of ADR monitoring and its importance. Attendees were encouraged to report all suspected ADRs using various reporting modalities, such as using a printed ADR notification form (available at all nursing stations), reporting ADRs by telephone, or directly reporting ADRs to an attending clinical pharmacist in certain hospital departments. Nurses also completed notification forms. Many forms were designed for this study, including a notification form and a form to describe the ADR in detail. Notification forms were kept in the participating wards. All patients were assessed for ADRs during the study period. In suspected cases, patients’ past medical history and medication history were collected. To provide complementary information concerning adverse reactions, especially unexpected reactions, ADRs were spontaneously reported as part of standard care. Patients were monitored daily throughout their hospital stay, and some their medical records were reviewed daily and others after discharge. The suspected ADRs were carefully analysed and documented. All relevant data, including all drugs that patients received prior to the reaction onsets and their respective dosages, the most frequent routes of administration, the dates of the reaction onsets, and the patients’ allergy status (to drugs and foods), were noted. Thus, the ADRs confirmed by the physicians and research pharmacists were classified and subjected to a severity assessment. Furthermore, information regarding the ADRs reported in the unit was published six times a year in a news bulletin of the Department of Pharmacovigilance (PHS) and was disseminated to all health care professionals at the hospital.

After initial notification of a suspected ADR, additional details were collected concerning previous allergies, concomitant medications, comorbidities, ADR management and outcome, and other details necessary for evaluation. These data were collected by reviewing patients’ records and noting the reporters’ comments. The collected data were recorded in separate ADR documents for further assessment. The physician responsible for the case was consulted when additional details and clarification were necessary.

### Evaluation of data

#### Patient characteristics

The patients’ age and gender were considered in the evaluation. In accordance with a previous paper, patients were divided into three age groups: children and teenagers (0–18 years old), adults (19–59 years old), and the elderly (over 60 years old).

#### Reaction characteristics

The ADRs were classified according to the Rawlins and Thompson classification system as type A or type B [17]. The severity of the reaction was determined according to the classification system of Hartwig et al. [18-20]. Mild reactions were those that were self-limiting, resolved over time without treatment, and did not extend a patient’s hospital stay. Moderate ADRs were defined as those that required therapeutic intervention and prolongation of the hospital stay by one day but that resolved within 24 hours due to a change in drug therapy or the administration of a specific treatment to prevent further adverse outcomes. Severe ADRs threatened patients’ lives, caused disability, led to hospitalisation, prolonged hospital stays, required intensive medical care, or led to death. Reactions were further classified depending on the organ system affected.

#### Drug characteristics

The drugs involved in the ADRs were categorised into various drug classes according to the anatomical therapeutic chemical (ATC) classification, based on the 2005 WHO-ATC Index [21].

#### Management and outcomes

Patients’ outcomes were reported as death, fully recovered (during hospitalisation), recovering (but not fully recovered during hospitalisation), or unknown (not documented after the initial report in the chart). The management strategies used for the ADRs were categorised as drug withdrawal, dose reduction, additional treatment for the ADR, or no change in regimen with no additional treatment.

#### Predisposing factors

Factors with the potential to predispose patients to ADRs in the individual reports were evaluated. Predisposing factors were generally classified according to age, gender, multiple and intercurrent disease states, and polypharmacy [3-8]. Ages above 60 (geriatrics) and below 18 (paediatrics) were regarded as a predisposing factor under the age criterion. Polypharmacy was considered to be minor (2–3 drugs), moderate (4–5 drugs), or major (>5 drugs) based on the characterisation by Wong [6]. Gender was considered a factor only if there was previous information indicating that the patient’s gender predisposed the patient to the reaction in question.

#### Statistical analysis

Student’s t-test was used to compare means, and the χ^2^ test was used for the other variables. A two-tailed P value of less than 0.05 was considered statistically significant.

### Ethics and consent

The study was approved by the Institutional Human Ethics Committee of Centro Universitário Luterano de Palmas-Tocantins, filed under number 794/2008, and was conducted in accordance with the ethical guidelines of the Declaration of Helsinki (created in 1964 and revised in 2002). Permission to conduct the study was obtained from the Medical Superintendent of the Hospital Geral de Palmas.

## Results

Throughout the 8-month study period, 95 ADRs were confirmed and reported in 81 inpatients. There were 2995 patient admissions at locations other than HGP, and the overall incidence of ADRs during hospitalisation in this patient group was 3.1%.

ADRs were more frequent in males (55.7%) than in females (44.3%). No significant difference was observed in the ADRs between males and females during the hospital stay (χ^2^c = 1.05, P = 0.26). The rates of ADRs in paediatric (<18 years), geriatric (>60 years), and adult patients were 18.9, 20.0, and 61.0%, respectively. The rates of ADRs in adult patients were significantly higher than those in paediatric and geriatric patients (χ^2^ = 33; P = 0.0001). Details regarding the classification and assessment of ADRs are provided in Table 1.

**Table 1:** Patient characteristics

**Age group**	**Number (%) of ADR reports**	**Gender group**	**Number (%) of ADR reports**
	**(n = 95)**		**(n = 95)**
Paediatric (0–18 years)	18 (19.0)	Male	53 (55.7)
Adult (19–59 years)	58 (61.0)	Female	42 (44.3)
Geriatric (>60 years)	19 (20.0)		
**Total**	95 (100.0)		95 (100.0)

Certain factors contributed to the occurrence of ADRs, such as the number of different drugs administered concomitantly. To assess contributing factors, we calculated the median number of prescriptions per patient that were suspected of causing ADRs, which was 6.8 medications/prescription. We also found that the risk of ADRs was higher in 7.4% of the patients who were using more than 6 medications.

The severity assessment of the ADRs showed that over half of the reactions reported were moderate (58.9%) (χ^2^ = 29.3, P = 0.00001), followed by mild (25.3%) and severe (15.8%) reactions. There were no fatal reactions. Complete recovery was achieved in 26.3% of patients with ADRs, 5.8% were in the recovery process, and 57.9% were classified as having ‘unknown outcomes’ (i.e., outcomes that could not be assessed due to a lack of recorded reports). Treatment with the offending drug was interrupted in 50.5% of patients. Another drug was substituted for the offending drug in 27.4% of patients, and other drugs were added to relieve the symptoms in 57.0% of patients; the drug dosage was not reduced in any patient to ameliorate symptoms. Treatment was unchanged in 37.8% of patients (Table 2). The majority of reported reactions were type A reactions (82.1%).

**Table 2:** Management, outcomes, and severity of ADRs

**Parameters**	**Number (%) of ADRs**
**Severity**	
**Mild**	24 (25.3)
**Moderate**	56 (58.9)
**Severe**	15 (15.8)
**Outcomes†**	
**Fatal**	0
**Fully Recovered**	25 (26.3)
**Recovering**	15 (15.8)
**Unknown**	55 (57.9)
**Treatment**	
**Stopped the medication**	48 (50.5)
**Reduced the dose**	0
**Added another drug to relieve the symptoms**	54 (57.0 )
**Substituted another drug**	26 (27.4)
**No change**	36 (37.8 )

The most common drugs causing ADRs and their reaction details are shown in Table 3. Analgesics (e.g., metamizole) were associated with approximately one-third of all ADRs reported (21.0%). Tramadol produced the highest number of reactions (11.6%), followed by vancomycin (8.4%), phenytoin (6.3%), and ceftriaxone (4.1%). Itching was the most common ADR reported (26.3%), followed by rashes and oedema (13.7%). The organ systems affected by the ADRs are shown in Table 4. The skin was found to be the most commonly affected organ system (34.5%), followed by the metabolic (16.5%) and gastrointestinal (14.2%) systems.

**Table 3:** Drug classes and individual drugs most commonly associated with ADRs

**Drug class**	**Drug (ATC)**	**Total number (%)**
		**(n = 95)**
Antibiotics	Cefalexin (J01DB01)	1 (1.0)
	Cefalotin (J01DB03)	6 (6.3)
	Cefazolin (J01DB04)	1 (1.0)
	Cefepime (J01DE01)	3 (3.1)
	Ceftriaxone (J01DD04)	4 (4.2)
	Imipenem (J01DH51)	2 (2.1)
	Oxacillin (J01CF04)	1 (1.0)
	Rifampicin (J04AB02)	1 (1.0)
	Vancomycin (J01XA01)	8 (8.4)
Analgesics	Metamizole (N02BB02)	20 (21.0)
	Paracetamol (N02BE01)	1 (1.0)
Antipsychotics	Chlorpromazine (N05AA01)	1 (1.0)
	Olanzapine (N05AH03)	1 (1.0)
	Risperidone (N05AX08)	1 (1.0)
Opioids	Fentanyl (N01AH02)	1 (1.0)
	Tramadol (N02AX02)	11 (11.6)
Benzodiazepine	Diazepam (N05BA01)	1 (1.0)
	Midazolam (N05CD08)	1 (1.0)
ACE inhibitors	Captopril (C09BA01)	1 (1.0)
	Enalapril (C09AA02)	2 (2.1)
Antiarrhythmic	Amiodarone (C01BD01)	2 (2.1)
Local anaesthetic	Bupivacaine (N01BB01)	1 (1.0)
Anticonvulsant	Phenytoin (N03AB02)	6 (6.3)
Beta-blocker	Carvedilol (C07AG02)	1 (1.0)
Antiemetic	Metoclopramide (A03FA01)	2 (2.1)
H2 receptor antagonist	Ranitidine (A02BA02)	2 (2.1)
Antidiuretic	Furosemide (C03CA01)	4 (4.2)

**Table 4:** Organ systems affected by ADRs and the most commonly reported reactions

**Organ system**	**Number (%) of ADRs**
	**(n = 133)**
Skin	46 (34.5)
Gastrointestinal	19 (14.2)
Central nervous system	12 (9.0)
Cardiovascular	5 (3.7)
Eyes, ears, nose, and throat	3 (2.2
Musculoskeletal	9 (6.7)
Metabolic	22 (16.5)
Haematologic	4 (3.0)
Respiratory	2 (1.5)

The minimum amount of time prior to ADR development was 1 day of hospitalisation, the median time was 6 days, and the maximum time was 180 days. The reactions that manifested during a period of 11 to 30 days of hospitalisation constituted 31.6% of the total number of reactions identified in this study, equivalent to the average percentage of reactions that occurred among inpatients in the Orthopaedics and General Medicine Departments.

The highest ADR rate in young adults occurred in the Orthopaedics Department (25%). ADRs occurred in the General Medicine (22%) and Oncology (16%) Departments at the second and third greatest frequencies, respectively.

## Discussion and conclusion

A total of 30-91% of ADRs could be avoided, thus saving health system resources and reducing harm to patients [22]. To work together toward ADR prevention, physicians, nurses, and pharmacists should be aware of potential clinical problems by assessing medicines that a patient has used recently; allergies or unusual reactions to any medicine, food, or product; special dietary or eating restrictions; and whether the patient is pregnant, breast-feeding, or planning pregnancy in the near future [23]. New drugs should also be closely monitored to avoid unknown and severe ADRs [24].

The fundamental role of pharmacovigilance centres is to collect and process data regarding ADRs and to support hospitals in the identification of these reactions [25]. The centres’ actions serve to reduce risks related to medication usage, improve patients’ quality of life, prevent iatrogenic diseases, and minimise health expenses.

The frequency of ADRs found in this study, which was based on inpatient records, was 3.1%. This value could have been greater if HGP had adopted intensive monitoring techniques or computer programs to supervise ADRs. The main reason for this low number is that our data were derived from spontaneous reporting. The ADR rate was low compared with the results of a meta-analysis conducted by Lazarou et al. [12], who reported that 15.1% of hospitalised patients develop an ADR. Other factors that may have contributed to this low number include the non-reporting of mild ADRs and the lack of guidelines and procedures for identification, registration, and notification. Reluctance to register ADRs persists, especially among nurses, as the registering of ADRs could signal medical mistakes or poor quality of care. This reluctance results in fewer ADR notifications and was also observed in Sobravime’s study [13].

There is no agreement among studies regarding the incidence of ADRs with respect to gender. Certain authors [3,4] have reported that women are more susceptible to ADRs, possibly due to their high medication use, obstetric complications, and metabolic alterations due to hormone levels. Other researchers [26-28] have found the incidence of ADRs to be unrelated to gender, which supports our finding that ADRs did not differ significantly between men and women.

Age is considered a risk factor for the occurrence of ADRs [29]. Therefore, children and the elderly, due to metabolic system alterations, require careful orientation and follow-up to avoid ADR occurrences and complications. However, in our study, the incidence of ADRs in adults (61.0%) was significantly higher than that in the other age groups [26,30]. These results seem to contradict those of Passarelli [4], who found that the elderly have a higher risk of ADRs. These conflicting results may be due to the dosage adjustments of paediatric and geriatric prescriptions as well as the higher number of young adults who are hospitalised at HGP.

The incidence of adverse reactions increases exponentially, but not necessarily simultaneously, with the number of drugs administered during a certain period [6]. Our study demonstrated that the usage of 6 to 10 medications per patient increased the risk of ADRs in 7.4% of cases.

Extension of hospital stay is also considered a risk factor for ADRs [26]. The highest ADR frequency (approximately 50%) occurred during the first five days of hospitalisation. The minimum time prior to the development of ADRs was 1 day of hospitalisation, the median time was 6 days, and the maximum time was 180 days. The percentage of reactions that manifested during a period of 11 to 30 days of hospitalisation was 31.6%, which is equivalent to the average percentage of reactions among inpatients of the Orthopaedics and General Medicine Departments at HGP.

The higher ADR rate in young adults is related to the higher ADR frequency in the Orthopaedics Department (25%). These results revealed a correlation between the elevated number of young adults hospitalised and the number of traumas caused by motorcycle accidents, which are the second most common cause of hospitalisation at HGP. The second and third highest prevalence of ADRs occurred in the General Medicine (22%) and Oncology (16%) Departments, most likely due to the greater exposure to medication in the General Medicine Department and to the adverse effects of antineoplastic medications in the Oncology Department.

It was observed that several organ systems were affected by medications. However, in accordance with other studies [4,31], the highest frequency (48.4%) of adverse reactions occurred in the dermatological system, manifesting as formication, skin rashes, flushing, and dried skin. Eleven reactions were registered simply as allergic reactions with no further description in the records regarding the associated reaction manifestation, compromising the quality of the records and the notification process.

In our study, analgesics caused the highest rate of ADRs, followed by antibiotics. Our results are in accordance with those of a study by Bates [32], possibly due to the elevated consumption of such medications at HGP. Risperidone, olanzapine, ceftriaxone, vancomycin, and furosemide were responsible for severe ADRs (type B); although the medications’ dose dependence and pharmacological properties were not indicated in the reports, severe ADR cases are frequently immune- or genetically related and usually prolong hospitalisation or require follow-up treatment. However, the majority of the ADRs studied were type A reactions (84.2%).

Due to intervention, the majority of ADRs were of moderate severity, and there was a significant difference in the degree of severity of the ADRs based on the records analysed.

A few doctors were able to control the ADRs by discontinuing the offending medication (50%). In other cases, clinical treatments were implemented using antihistamines, corticoids, antidotes, zinc dioxide, and vitamin creams to relieve symptoms, whereas no treatment was administered in some cases either due to the presence of only a mild ADR or because the offending medication was unknown (13%).

In conclusion, the ADRs that occurred at our hospital are comparable to those reported by other studies performed in Brazilian and foreign hospitals; nevertheless, certain aspects were different. The number of prescribed drugs and the length of drug use constitute risk factors for ADRs; monitoring these factors requires a review of clinical protocols, the quality of the prescription, and the therapeutic arsenal. Among the prescribed medications, metamizole, tramadol, and vancomycin caused the most ADRs due to their frequent usage and the inherent characteristics of these drugs. It is evident that pharmacovigilance systems are needed to facilitate ADR follow-ups by health professionals directly involved in patient care.

## Competing interests

The authors have no competing interests to declare.

## Authors’ contributions

MGAAL and MCSP participated in the design of the study, analysed the data, and drafted the manuscript. SMBP analysed the data. JGDC participated in the design of the study and analysed the data. MCSP and VGM conceived the study. All authors read and approved the final manuscript.

## Pre-publication history

The pre-publication history for this paper can be accessed here:

http://www.biomedcentral.com/2050-6511/14/5/prepub

## References

[B1] World Health Organization International drug monitoring: the role of the hospital Technical report series no. 425 1966 Geneva, Switzerland: World Health Organization 1 24 4981079

[B2] Juntti-Patinen L Neuvoren PJ Drug-related deaths in a university central hospital Eur J Clin Pharmacol 2002 58 479 482 10.1007/s00228-002-0501-2 12389071

[B3] Magalhães SMS Carvalho WS Reações Adversas a Medicamentos Ciências Farmacêuticas. Uma Abordagem em Farmácia Hospitalar 2001 São Paulo: Atheneu 125 146

[B4] Passarelli MCG Reações adversas a medicamentos em uma população idosa hospitalizada. PhD thesis 2005 Universidade de São Paulo, Faculdade de Medicina

[B5] Lobstein R Lakin A Koren G Pharmacokinetic Changes during pregnancy and Their clinical Relevance Clinical Pharmacokinet 1997 33 328 343 10.2165/00003088-199733050-00002 9391746

[B6] Wong A Os usos inadequados e os efeitos adversos de medicamentos na prática clínica J Pediatr (Rio J) 2003 79 379 380 14557834

[B7] Tatro DS Textbook of therapeutics, drug and disease management 1996 Baltimore: William and Wilkins

[B8] May RJ Adverse drug reactions and interactions Pharmacoterapy: a pathophysiologic approach 1997 Norwalk: Appleton & Lange 101 116

[B9] Laporte JR Famacovigilância Hospitalarhttp://www.cvs.saude.sp.gov.br/farde_hos.html

[B10] Brandão A Vasconcelos F A tênue fronteira entre a cura e o malefício Pharmacia Brasileira 2000 22 36 39

[B11] Einarson TR Drug-related hospital admissions Ann Pharmacother 1993 27 832 840 8364259 10.1177/106002809302700702

[B12] Lazarou J Pomeranz BH Corey PN Incidence of adverse drug reactions in hospitalized patients: A meta-analysis of prospective studies JAMA 1998 279 1200 1205 10.1001/jama.279.15.1200 9555760

[B13] Sobravime Declaração de Berlim sobre Farmacovigilância 2005 São Paulo

[B14] Van Grootheest K Olsson S Couper M de Jong-van den Berg L Pharmacists’ role in reporting adverse drug reactions in an international perspective Pharmacoepidemiol Drug Saf 2004 3 457 464 15269929 10.1002/pds.897

[B15] Gallelli L Ferreri G Colosimo M Pirritano D Flocco MA Pelaia G Retrospective analysis of adverse drug reactions to bronchodilatorsobserved in two pulmonary divisions of Catanzaro, Italy Pharmacol Res 2003 47 Suppl 6 493 499 12742002 10.1016/s1043-6618(03)00003-3

[B16] Wu WK Evaluation of outpatient adverse drug reactions leading to hospitalization Am J Health Syst Pharm 2003 60 Suppl 3 253 259 12613234 10.1093/ajhp/60.3.253

[B17] Rawlins MD Thompson JW Mechanisms of adverse drug reactions Textbook of adverse drug reactions 1981 4 Oxford: Oxford University press 18 45

[B18] Naranjo CA Busto U Sellers EM Sandor P Ruiz I Robert EA Ecek E Domeck C Greenblatt DJ A method for estimating the probability of adverse drug reactions Clinic Pharmacological Therapeutic 1981 2 239 245 10.1038/clpt.1981.154 7249508

[B19] Pearson TF Pittman D Longley JM Grapes T Vigliotti DJ Mullis SR Factors associated with preventable adverse drug reactions Am J Hosp Pharm 1994 51 2268 2271 7801987

[B20] Sebastião ECO Intervenção farmacêutica na qualidade assistencial e nas reações adversas de amitriptilina prescrita para pacientes ambulatoriais do Sistema Único de Saúde de Ribeirão Preto. PhD thesis 2005 Universidade de São Paulo, Faculdade de Ciências Farmacêuticas

[B21] WHO Collaborating Centre for Drug Statistics Methodology: Completed ATC Index 2005 [http://www.whocc.no/atcddd/]

[B22] Puerro MAP O custo das reações adversas medicamentosas em hospitais MSc thesis 2004 Universidade de São Paulo, Faculdade de Saúde Pública

[B23] Merck Sharp Dohme Aspectos gerais dos medicamentos Manual Merck. Saúde para a família Seção 2 – Medicamentos. Capítulo 5 [http://mmspf.msdonline.com.br/pacientes/manual_merck/secao_02/cap_005.html]

[B24] Harnik S Respostas nocivas a medicamentos podem ser evitadas, diminuindo custos[http://www.usp.br/agen/bols/2004/rede1426.htm]

[B25] Gomes MJV Reis AAMM Ciências Farmacêuticas: Uma Abordagem em Farmácia Hospitalar 2001 São Paulo: Atheneu

[B26] Rozenfeld S Agravos provocados por medicamentos em hospitais do Estado do Rio de Janeiro Rev Saude Publica 2006 41 1 8 10.1590/s0034-89102007000100015 17273641

[B27] Gomes AP Incidência de reações adversas a medicamentos em Hospital de Ensino do Nordeste do Brasil. MSc thesis 2004 Universidade Federal do Ceará, Faculdade de Ciências Farmacêuticas

[B28] Pfaffenbach G Carvalho OM Bergsten-Mendes G Reações adversas a medicamentos como determinantes da admissão hospitalar Rev Assoc Med Bras 2002 48 237 241 10.1590/S0104-42302002000300037 12353108

[B29] Notteerman DA Farmacoterapia Pediátrica Farmacologia em Terapia Intensiva 1993 Rio de Janeiro: Revinter 96 120

[B30] Louro EO Perfil de utilização de medicamentos e monitoração de reações adversas em pacientes pediátricos no Hospital Albert Sabin em São Paulo Rev Saude Publica 2004 41 23 29

[B31] Amaral FPM Avaliação do sistema de farmacovigilância do instituto de doenças tropicais Natan Portella em Teresina – PI. MSc Thesis 2006 Universidade Federal do Ceará, Faculdade de Medicina

[B32] Bates DW Cullen DJ Laird N Incidence of adverse drug events and potential adverse drug events JAMA 1995 274 29 34 10.1001/jama.1995.03530010043033 7791255

